# Unveiling the Therapeutic Spectrum of *Annona reticulata* Linn.: Antibacterial, Insecticidal, and Antidiarrheal Insights

**DOI:** 10.1155/tswj/9100797

**Published:** 2026-04-01

**Authors:** Tasnia Binte Bari Kabbo, Md. Sohel Rana, Goutam Kumar Roy, Pritesh Ranjan Dash

**Affiliations:** ^1^ Department of Pharmacy, Jahangirnagar University, Savar, Dhaka, Bangladesh, juniv.edu; ^2^ Department of Pharmacy, ASA University Bangladesh, Shyamoli, Dhaka, Bangladesh, asaub.edu.bd

**Keywords:** *Annona reticulata* Linn., antibacterial activity, antidiarrheal property, extract, insecticidal activity, phytochemistry

## Abstract

The focus of recent research has been on developing natural medicines. Natural substances of the *Annona reticulata* Linn. plant, which belongs to the *Annonaceae* family, have medicinal uses. The antibacterial, insecticidal, and antidiarrheal properties of *Annona reticulata* Linn. methanol and aqueous leaf fractions were assessed in this study along with phytochemical screening and GC–MS analysis. Both extracts’ antibacterial properties were evaluated at a dosage of 250 mg/mL using the disc diffusion method. While the zones of inhibition values for aqueous extract were lower than those for methanol extract, the zones of inhibition values for methanol leaf extract against specific gram‐positive and gram‐negative bacterial strains were almost equivalent to those obtained from the standard drug tetracycline hydrochloride. The methanol extract showed strong insecticidal potential against *T. castaneum* and *P. americana*, which was found to be even more effective than the standard permethrin solution. Additionally, the aqueous leaf extract showed good insecticidal activity; however, it was less effective than the methanolic extract. Castor oil–induced diarrhea test was utilized for assessment of antidiarrheal potentials; at dosages of 200 and 400 mg/kg of body weight, methanol and aqueous fractions showed significant outcomes with notable onset of diarrhea and total quantity of feces values (*p* < 0.001). Both methanol and aqueous leaf fractions demonstrated percentage inhibition of diarrhea values of 44.19% and 41.86%, respectively, at a 400 mg/kg dosage. In the enteropooling test, the castor oil–induced intestinal volume was also significantly (*p* < 0.001) decreased by both methanol and aqueous extracts. Furthermore, intestinal transit was inhibited by aqueous and methanol extracts by 50.89% and 52.78%, respectively, at a 400 mg/kg dosage in GI motility tests. The outcomes significantly resembled those of the standard drug loperamide (5 mg/kg). Plant extracts were screened for certain phytochemicals using a preliminary phytochemical screening assay. GC–MS analysis was utilized for the determination of the chemical constituents that were responsible for achieving these activities.

## 1. Introduction

Natural products have a long history of producing amazing secondary metabolites that resemble drugs to treat a wide range of illnesses that still affect people today [1]. The capacity to eliminate or stop the growth of germs in natural, semisynthetic, or synthetic chemicals at levels that can be achieved in vivo is known as antimicrobial activity [2]. The most effective treatment for infectious ailments nowadays is antibiotics. According to Cheng et al., the absence of newly discovered antimicrobial medications and the development of antibiotic resistance seriously threaten human and animal health [3]. Due to the shown effectiveness of traditional treatment, efforts to find novel chemotherapeutic options to eradicate drug‐resistant bacterial infections and lessen the negative effects of antibiotics have been made [4, 5]. Numerous investigations conducted worldwide have demonstrated numerous antibacterial potentials of plants and their extracts [6]. Pesticides, genetically modified plants, cultural techniques, host plant resistance, biological control, and microbial management are some of the tools and strategies that can be used to manage pest insects [7]. However, insecticides are essential to the management of insects in modern agriculture. Unfortunately, most synthetic pesticides have a number of negative side effects and many insects are no longer susceptible to chemical insecticides. Therefore, finding novel pesticides with distinct mechanisms of action, enhanced selectivity against target insects, and answers to human safety concerns is critically important. Historically, botanical pesticides have been preferred over synthetic ones for controlling insect pests because of their minimal environmental and public health risks. In addition, for farmers in underdeveloped nations, plant extracts are safe, affordable, simple to prepare, and effective [8, 9]. A decrease in stool shape (increased looseness of stool), along with more frequent and fluid bowel movements per day, are signs of diarrhea, a common gastrointestinal ailment. Even though some antibiotics are used to treat diarrhea, bacteria often get resistant to them and they frequently have negative side effects. Therefore, one of the main goals of current research has been to find safer and more effective antidiarrheal medications from plants [10, 11]. Moreover, the technique of using a straightforward and affordable assay to evaluate and identify the chemical compounds present in plants, particularly the secondary metabolites, is known as phytochemical screening [12]. Furthermore, the process of identifying distinct components in a sample is known as GC–MS [13]. In addition, this method essentially uses the properties of both mass spectrometry and gas chromatography to detect distinct molecules in a given sample based on retention time and a specific fragmentation pattern of a compound [14]. One notable source of potential therapeutic chemicals is the *Annona* genus of plants (*Annonaceae* family). It is endemic to tropical and subtropical areas. Folk medicine has employed this plant’s parts, such as leaves, bark, seeds, and roots, to treat a variety of ailments [15]. The antibacterial, insecticidal, and antidiarrheal potentials of aqueous and methanol fractions from *Annona reticulata* Linn. leaves were investigated via this study, along with phytochemical screening and GC–MS analysis. Although previous studies have reported the antibacterial, insecticidal, and antidiarrheal activities of *Annona reticulata* Linn., most of those investigations focused on individual bioactivities without establishing a comprehensive comparative evaluation using the same plant source, extraction conditions, and experimental framework. Moreover, limited attention has been given to correlating these biological effects with the probable phytochemical constituents responsible for such activities. Therefore, the present study was designed to provide a combined assessment of antibacterial, insecticidal, and antidiarrheal potentials of methanolic and aqueous leaf extracts of *Annona reticulata* Linn. under the same experimental conditions. In addition, this investigation attempts to link the observed bioactivities with the presence of probable bioactive phytochemicals, thereby offering a more systematic understanding of the antibacterial, insecticidal, and antidiarrheal properties of the plant.

## 2. Materials and Methods

### 2.1. Preparation of Plant Extracts

*Annona reticulata* Linn. leaves were gathered from Savar, Dhaka, Bangladesh. To prepare it for grinding, the plant material (leaves) was first sun‐dried. After that, drying at a lower temperature was conducted in a Gallenkamp oven. Then, using a grinding mill, plant parts were pulverized into powders. 538 and 630 gm of processed dry plant material were placed in dark‐colored flasks, sealed with 5.4 L of water and 6.3 L of methanol (as solvent), and kept at room temperature. For 15–20 days, the plant parts were stored in sealed containers, shaken, and stirred periodically. After that, the materials were filtered by utilizing cotton bed filters and then lastly, with Whatman No. 1 filter papers. To achieve concentration of sticky crude extracts, the filtrates were dried at 40 ± 2°C in rotary evaporator. The materials were properly labeled after extraction and kept in sterile sample containers at 4°C [16]. The percentage yield of the extracts was calculated based on the initial dry weight of the plant material. The methanolic extract yielded 12.7% (w/w), while the aqueous extract yielded 14.5% (w/w).

### 2.2. Phytochemical Screening Test

#### 2.2.1. Test for Carbohydrates, Glycosides, Alkaloids, Saponins, Flavonoids, Tannins, Steroids, Glucosides

Utilizing standard methodologies and reagents, the existence of these phytochemicals was examined [17–22].

### 2.3. GC–MS Analysis of Aqueous and Methanol Extracts From *Annona reticulata* Linn. Leaves

GC–MS analysis was performed using the methodology outlined in our earlier article [23].

### 2.4. In Vitro Assessment of Antibacterial Property

#### 2.4.1. Collection of Test Microorganisms

The Microbiology Department of Jahangirnagar University provided the bacterial strains of four gram‐positive origin (*Bacillus subtilis, Staphylococcus aureus, Mycobacterium tuberculosis, Listeria monocytogenes*) and three gram‐negative origin (*Escherichia coli, Vibrio cholerae, Klebsiella pneumoniae*). *Mycobacterium tuberculosis* H37Ra, an avirulent laboratory strain, was included in this study to evaluate the potential of the extracts against a pathogen of significant global health concern. All experiments involving this organism were conducted following appropriate biosafety guidelines under controlled laboratory conditions in a certified BSL‐3 facility to ensure safe handling.

#### 2.4.2. Preparation of Media

To make double‐strength nutritional media for antibacterial investigation, peptone (1 g), yeast (0.3 g), and sodium chloride (0.5 g) were submerged in water (50 mL). After that, the media were autoclaved for 15 min at 15 lb/psi to sterilize them. After chilling and filtering, the pH was lowered to 5.4 with the addition of 10% lactic acid. Autoclaving the media for 15 min at 15 lb/psi pressure sterilized it [24].

#### 2.4.3. Disc Diffusion Method for Evaluation of Antibacterial Activity

Particular bacterial cultures (0.1 mL) were added to nutrient agar medium (30 mL) on petri dishes in order to evaluate the crude fractions. The bacterial inoculum was prepared from 18‐ to 24‐h old cultures and adjusted to a 0.5 McFarland turbidity standard (approximately 1.5 × 10^8^ CFU/mL) using sterile normal saline prior to inoculation. Crude plant extracts generally require higher test concentrations compared to purified antibiotics due to the presence of a complex mixture of active and inactive constituents, which may reduce diffusion efficiency in agar media. Therefore, a concentration of 250 mg/mL was selected to ensure detectable antibacterial properties during the preliminary screening using the disc diffusion method. Whatman No. 1 papers were soaked in beakers filled with dissolved leaf fractions (250 mg/mL). After being removed with sterile forceps, the paper discs were then set out on plates containing various species and left to air dry. Incubation of the plates (37°C, 24 h) was conducted. Zones of inhibition were measured in millimeters, and antibacterial activity was assessed by comparing the inhibition zones against the test microorganisms. All experiments were performed in triplicate (*n* = 3) on independently prepared agar plates, and the results were expressed as mean ± SD to minimize plate‐to‐plate variability. As a reference standard and negative control, tetracycline hydrochloride (1 mg disc) and DMSO were used. Both the reference and the negative control were utilized to compare the growth inhibition [25].

#### 2.4.4. Determination of Minimum Inhibitory Concentration (MIC)

To ascertain MIC, the microdilution broth method was employed. The MIC of methanol and aqueous leaf fractions from *Annona reticulata* Linn. that inhibited the growth of bacteria was analyzed. 250 mg/mL of the plant extract was serially diluted with DMSO to create 250, 125, 62.5, 31.25, 15.625, and 7.813 mg/mL. Inoculation of the wells with 0.1‐mL aliquots of test organisms (10^6^ cfu/mL) was done utilizing serial dilutions of test extracts (50 μL, each). The microplate was incubated for twenty‐four hours at 37°C ± 1°C. All MIC determinations were performed in triplicate (*n* = 3). Each assay included a growth control (broth with bacterial inoculum but without extract) and a sterility control (broth only). The MIC was determined by dilution of the test extracts corresponding to the appropriate test organism that did not exhibit observable growth [25].

### 2.5. In Vivo Studies

#### 2.5.1. Experimental Animals

In order to examine insecticidal properties, adult *T. castaneum* were gathered from infested stored grains in a warehouse located in Savar, Dhaka, whereas adult *P. americana* were collected from the pharmacy department’s storage rooms at Jahangirnagar University. The placement of both insects was done in separate holes containing plastic containers; the holes allowed the passage of air. In addition, the department’s animal home provided the Wister Albino rats of both sexes used in the investigation of antidiarrheal activity, which weighed between 120 and 230 g. In Jahangirnagar University’s Pharmacology Laboratory, methanol and aqueous leaf extracts of *Annona reticulata* Linn. were investigated pharmacologically. The animals were housed under standard laboratory conditions with controlled temperature (22 ± 2°C), relative humidity (55 ± 10%), and a 12 h light/dark cycle in cages made of polypropylene. Rats were acclimatized to the laboratory environment for 7 days prior to the experiments. Pelletized mouse feed from ICDDR,B was given to the rats, and they were allowed unlimited access to water. All experimental procedures involving animals were conducted in accordance with the ARRIVE guidelines and followed the OECD rules for the care and use of laboratory animals for toxicity and pharmacological studies. The study protocol was reviewed and approved by the Animal Ethics Committee of Jahangirnagar University (Ref No: BBEC, JU/M 2024/11 (137)). During experimental procedures, animals were handled gently to minimize stress. At the end of the study, animals were humanely euthanized using an overdose of anesthetic agents in accordance with internationally accepted ethical standards.

#### 2.5.2. In Vivo Evaluation of Insecticidal Activity

The insecticidal bioassay was designed in accordance with relevant WHO recommendations for laboratory evaluation of insecticides and adapted principles from OECD test guidelines for toxicity assessment. Adult *T. castaneum* and *P. americana* were collected, and prior to experimentation, insects were acclimatized for at least 7 days under controlled environmental conditions: temperature 27 ± 2°C, relative humidity 65 ± 5%, and a 12:12 h light–dark cycle. Only healthy, active insects of similar age and size were used in the assays. The assay was conducted using the filter paper contact method following the WHO‐recommended procedures with minor modifications. Mortality was recorded at predefined exposure intervals following continuous contact with treated filter papers. Standard permethrin (positive control), aqueous, and methanolic leaf extracts were prepared at five concentrations: 1, 5, 10, 50, and 100 mg/2 mL. Methanol‐treated filter papers served as the negative control. All solutions were freshly prepared on the day of the experiment. Two milliliters of each test concentration were uniformly applied to Whatman No. 1 filter papers placed in sterile petri dishes. Treated filter papers were air‐dried at room temperature for 24 h to ensure complete solvent evaporation. Separate sets of petri dishes were prepared for each insect species and each concentration. Insects were gently introduced into the treated petri dishes and maintained under the same controlled environmental conditions. Twenty mature *T. castaneum* and 10 adult *P. americana* were put in each petri dish, and the insects’ motility, behavior (i.e., whether the drug attracts or repels them), and death were recorded at regular intervals. Insects were considered dead if they showed no movement upon gentle probing. The percentage mortality was calculated from the obtained data. Dose–response relationships were analyzed to determine median lethal dose (LD_50_) values by utilizing probit analysis. Probit analysis is a statistical technique used to determine the median lethal dose by transforming dose–response data into a linear regression line [26, 27].

#### 2.5.3. In Vivo Assessment of Antidiarrheal Property

##### 2.5.3.1. Castor Oil–Induced Diarrheal Method in Rat Model

With slight adjustments, the approach outlined by Asrie et al. was applied to examine the antidiarrheal potential of methanol and aqueous extracts from *Annona reticulata* Linn. leaves. Initially, six groups of five rats each were put together. After that, each rat was weighed and assigned a tail number. Normal saline (2 mL/kg) and loperamide hydrochloride (5 mg/kg) were given to test animals in control Groups I and II via the oral route. Aqueous leaf extracts at doses of 200 and 400 mg/kg were administered to rats in Groups III and IV, respectively, while methanol leaf fractions at the same doses were administered to rats in Groups V and VI, respectively. The doses of the test extracts were selected based on an acute toxicity study [28]. To induce diarrhea, 1 mL of highly refined analytical‐grade castor oil was administered to test animals 1 h after dosages. Each rat was kept in a separate cage (absorbent paper underneath). It was carefully recorded when diarrhea first appeared. For 4 h after the castor oil was given, the total amount of feces that each rat evacuated was counted and noted every hour and the percentage inhibition of diarrhea was computed [12, 29–31].

##### 2.5.3.2. Castor Oil–Induced Enteropooling

The efficacy of aqueous and methanol extracts to inhibit fluid accumulation is ascertained by utilizing the castor oil–induced enteropooling test. In this case, both male and female rats were fasted for 18 h. Six groups (a total of five) were created from the rats that were chosen for this test. Group I (the control group) received normal saline (2 mL/kg), while Group II (the standard group) was given loperamide (5 mg/kg) orally. Groups III–IV were administered 200 and 400 mg/kg of body weight of AEAR, whereas MEAR was administered orally to Groups V–VI at 200 and 400 mg/kg of body weight dosages. After an hour, each animal in each group received 1 mL of castor oil orally. From the pylorus to the cecum, the small intestine was separated after sacrificing all rats 2 h later. A graduated tube containing the intestinal contents was utilized to measure its volume [12, 32].

##### 2.5.3.3. Gastrointestinal Motility Test

The assay was conducted using Rahman et al.’s and Mascolo et al.’s methodology. From the chosen rats, six groups (five in each) were created. To induce diarrhea, castor oil (1 mL) was initially administered to each group of rats orally. After one hour, Group I received saline (2 mL/kg), Group II was treated with the standard medication (loperamide 5 mg/kg of body weight), Groups III–IV were given AEAR (200 and 400 mg/kg of body weight), and Groups V and VI were administered MEAR (200 and 400 mg/kg of body weight), orally. Administration of 1 mL of charcoal meal (10% charcoal suspension in 5% gum acacia) was performed via oral route to each animal after an hour. After killing all of the animals an hour after charcoal meal administration, it was determined what proportion of the intestinal distance—from the pylorus to the cecum—the charcoal meal traveled [33].

To minimize observational bias, the assessment was performed in a blinded manner. Experimental units were assigned at random to treatment and control groups. Thus, randomization was carried out using a random allocation technique, and to lessen observer bias, outcome assessment was carried out without knowledge of treatment allocation. Both male and female animals were used in this study and were randomly assigned to experimental groups. No sex‐specific differences were observed in the study outcomes, and data were therefore analyzed collectively. In addition, humane endpoints were predefined and included significant body weight loss (> 15%), signs of severe distress, reduced mobility, or abnormal behavior, at which point animals were humanely euthanized. Moreover, the sample size of five animals per group was selected based on prior published studies using similar experimental models [12, 32].

For statistical analysis, SPSS Version 27 and one‐way ANOVA for Windows were employed. Statistical analysis was performed to determine overall differences among groups, and *p* values were used to assess statistical significance. Data are presented as mean ± SEM. *p* < 0.05 was considered statistically significant. In addition, significant differences are indicated in the figures and tables using asterisks.

## 3. Results and Discussion

### 3.1. Phytochemical Screening

Aqueous and methanol leaf fractions from *Annona reticulata* Linn. underwent phytochemical screening, which revealed the presence of several chemical groups, as shown in the following table (Table 1).

**TABLE 1:** Findings of qualitative phytochemical screening of aqueous and methanol extracts of the leaves of *Annona reticulata* Linn.

Name of components	Presence/absence in aqueous extract	Presence/absence in methanol extract
Carbohydrate	Present	Present
Reducing sugar	Present	Present
Combined reducing sugar	Absent	Absent
Glycoside	Present	Present
Alkaloid	Present	Present
Saponin	Absent	Absent
Flavonoid	Present	Present
Tannin	Present	Present
Steroid	Present	Present
Glucoside	Present	Present

Numerous phytoconstituents, including tannins, flavonoids, glucosides, alkaloids, steroids, glycosides, and carbohydrates (monosaccharides and reducing sugars), have been found to be present in *Annona reticulata* Linn. leaves in the investigation. Saponin and combined reducing sugar were not detected in either extracts. The extracts’ diverse pharmacological effects may be due to their phytoconstituents. Reportedly, all these compounds are reported to possess antibacterial potentials [34–42]. According to reports, carbohydrates, glycosides, alkaloids, flavonoids, tannins, steroids, and glucosides possess insecticidal properties [43–49]. Additionally, phytochemical screening revealed the presence of some phytochemicals with antidiarrheal properties in the test extracts, such as carbohydrate, glycoside, alkaloid, flavonoid, and tannin [50–54]. It should be noted that the phytochemical screening performed in this study was based on standard qualitative tests, which only indicate the possible presence or absence of major classes of phytochemicals. These assays do not provide precise identification, structural confirmation, or quantitative estimation of the compounds present. Therefore, the results should be interpreted as preliminary indications, and further advanced analytical techniques are required for definitive characterization of the bioactive constituents.

### 3.2. GC–MS Analysis Reports of Aqueous and Methanol Extracts of *Annona reticulata* Linn. Leaves

**FIGURE 1:**
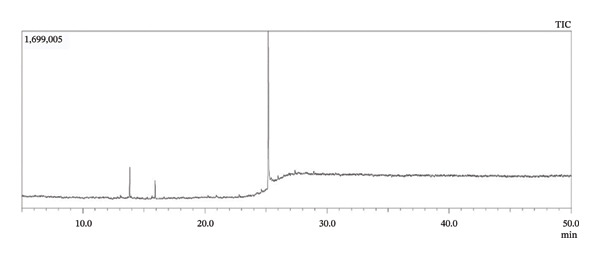
Total ionic chromatogram of *Annona reticulata* Linn. crude aqueous extract.

**FIGURE 2:**
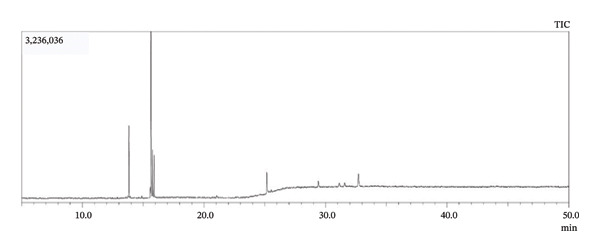
Total ionic chromatogram of *Annona reticulata* Linn. crude methanol extract.

As per the library search report, the crude aqueous extract of *Annona reticulata* Linn. leaves contained 77 different components, which are given in Table 2 (Figures 1 and 2).

**TABLE 2:** Constituents detected by GC–MS analysis in *Annona reticulata* Linn.’s crude aqueous extract.

Serial no.	Compound name	Molecular formula and molecular weight	Retention time	Area
1	Cyclopropyl carbinol	C_4_H_8_O; 72.10 g/mol	7.115	17,830
2	Thiophene‐3‐ol, tetrahydro‐, 1,1‐dioxide	C_4_H_8_O_3_S; 136.17 g/mol	7.115	17,830
3	Octodrine	C_8_H_19_N; 129.24 g/mol	7.115	17,830
4	1‐(5‐Bicyclo[2.2.1]heptyl)ethylamine	C_9_H_19_N; 141.25 g/mol	7.115	17,830
5	1‐Methyldecylamine	C_11_H_25_N; 171.32 g/mol	7.115	17,830
6	2‐Heptanamine, 5‐methyl‐	C_8_H_19_N; 129.23 g/mol	7.115	17,830
7	Benzeneethanamine, 2,5‐difluoro‐.beta.,3,4‐trihydroxy‐	C_8_H_12_F_2_NO_3_; 208.18 g/mol	7.115	17,830
8	Cystine	C_6_H_12_N_2_O_4_S_2_; 240.32 g/mol	7.115	17,830
9	Acetic acid, [(aminocarbonyl)amino]oxo‐	C_3_H_6_N_2_O_3_; 118.10 g/mol	7.115	17,830
10	4‐Fluorohistamine	C_5_H_8_FN_3_; 129.14 g/mol	7.115	17,830
11	Cyclobutanol	C_4_H_8_O; 72.10 g/mol	7.800	9329
12	1‐Methyldecylamine	C_11_H_25_N; 171.32 g/mol	7.800	9329
13	Pentanal	C_5_H_10_O; 86.13 g/mol	7.800	9329
14	D‐Alanine	C_3_H_7_NO_2_; 89.10 g/mol	7.800	9329
15	Eicosanoic acid, methyl ester	C_21_H_42_O_2_; 326.55 g/mol	15.908	56,805
16	Triacontanoic acid, methyl ester	C_31_H_62_O_2_; 466.81 g/mol	15.908	56,805
17	Docosanoic acid, methyl ester	C_23_H_46_O_2_; 354.60 g/mol	15.908	56,805
18	Tridecanoic acid, 12‐methyl‐, methyl ester	C_15_H_30_O_2_; 242.39 g/mol	15.908	56,805
19	Octadecanoic acid, 17‐methyl‐, methyl ester	C_20_H_40_O_2_; 312.52 g/mol	15.908	56,805
20	1,2‐Ethanediamine, N‐(2‐aminoethyl)‐	C_4_H_13_N_3_; 103.17 g/mol	16.803	15,238
21	2‐Methylaminomethyl‐1,3‐dioxolane	C_5_H_11_NO_2_; 117.15 g/mol	16.803	15,238
22	Alanine	C_3_H_7_NO_2_; 89.10 g/mol	16.803	15,238
23	1,2‐Ethanediamine, N‐methyl‐	C_3_H_10_N_2_; 74.13 g/mol	16.803	15,238
24	Propanamide	C_3_H_7_NO; 73.10 g/mol	18.061	22,359
25	2‐Propanamine, 1‐methoxy‐	C_4_H_11_NO; 89.14 g/mol	18.061	22,359
26	dl‐Alanine	C_3_H_7_NO_2_; 89.10 g/mol	18.061	22,359
27	Hydroxyurea	CH_4_N_2_O_2_; 76.06 g/mol	18.061	22,359
28	1‐Propanol, 2‐amino‐, (.+/−.)‐	C_3_H_9_NO; 75.11 g/mol	18.061	22,359
29	2‐Hexanamine, 4‐methyl‐	C_7_H_17_N; 115.22 g/mol	18.061	22,359
30	Ethanol, 2‐(methylamino)‐	C_3_H_9_NO; 75.11 g/mol	19.299	10,141
31	1,3‐Dioxolane‐4‐methanol	C_4_H_8_O_3_; 104.10 g/mol	20.130	5571
32	N‐dl‐Alanylglycine	C_5_H_10_N_2_O_3_; 146.15 g/mol	21.199	27,186
33	1‐Octadecanamine, N‐methyl‐	C_19_H_41_N; 283.53 g/mol	21.199	27,186
34	dl‐Alanyl‐dl‐norleucine	C_9_H_18_N_2_O_3_; 202.25 g/mol	21.199	27,186
35	(S)‐(+)‐1‐Cyclohexylethylamine	C_8_H_17_N; 127.23 g/mol	21.199	27,186
36	1‐Methyldecylamine	C_11_H_25_N; 171.32 g/mol	21.199	27,186
37	Benzeneethanamine, 2‐fluoro‐.beta.,3‐dihydroxy‐	C_8_H_10_FNO; 155.17 g/mol	21.199	27,186
38	Propanamide, N‐methyl‐2‐amino‐	C_4_H_9_N_2_O; 101.13 g/mol	22.100	5151
39	2‐Methylaminomethyl‐1,3‐dioxolane	C_5_H_12_NO_2_; 118.16 g/mol	22.845	33,954
40	Formamide, N,N‐dimethyl‐	C_3_H_7_NO; 73.10 g/mol	24.260	17,803
41	Propanediamide, 2‐amino‐	C_3_H_10_N_3_; 88.14 g/mol	24.260	17,803
42	L‐Alanine, TMS derivative	C_9_H_25_NO_2_Si_2_; 235.48 g/mol	24.260	17,803
43	1,3‐Dioxolane‐4‐methanol	C_4_H_8_O_3_; 104.10 g/mol	24.260	17,803
44	2‐(Methylamino)ethanol, O‐trimethylsilyl	C_6_H_18_NOSi; 148.30 g/mol	24.260	17,803
45	13‐Docosenamide, (Z)‐	C_22_H_43_NO; 337.57 g/mol	25.179	506,528
46	3‐Ethoxy‐1,1,1,5,5,5‐hexamethyl‐3‐(trimethylsilyl)trisiloxane	C_11_H_30_O_3_Si_4_; 322.71 g/mol	25.635	6749
47	Trisiloxane, 1,1,1,5,5,5‐hexamethyl‐3,3‐bis(trimethylsiloxy)	C_12_H_36_O_4_Si_5;_ 384.86 g/mol	25.635	6749
48	Heptasiloxane, 1,1,3,3,5,5,7,7,9,9,11,11,13,13	C_16_H_48_O_6_Si_7_; 533.17 g/mol	25.635	6749
49	2,4,6‐Cycloheptatrien‐1‐one, 3,5‐bis‐trimethylsilyl	C_13_H_24_OSi_2_; 252.50 g/mol	25.635	6749
50	Hexestrol, 2TMS derivative	C_24_H_40_O_2_Si_2_; 416.74 g/mol	25.635	6749
51	3‐Butoxy‐1,1,1,5,5,5‐hexamethyl‐3‐(trimethylsilyl)trisiloxane	C_13_H_36_O_3_Si_4_; 352.78 g/mol	25.635	6749
52	Methyltris(trimethylsiloxy)silane	C_10_H_30_O_3_Si_4_; 310.70 g/mol	25.635	6749
53	1‐Isopropoxy‐5‐propyl‐2,3‐bis(trimethylsilyl)benzene	C_18_H_38_OSi_2_; 326.66 g/mol	25.635	6749
54	Thymol, TMS derivative	C_13_H_22_OSi; 222.40 g/mol	25.635	6749
55	Silane, trimethyl[5‐methyl‐2‐(1‐methylethyl)phenyl]	C_13_H_24_Si; 208.41 g/mol	25.635	6749
56	Trimethylsilyl‐di(trimethylsiloxy)‐silane	C_9_H_27_O_2_Si_4_; 279.67 g/mol	26.430	21,459
57	3‐Isopropoxy‐1,1,1,5,5,5‐hexamethyl‐3‐(trimethylsilyl)trisiloxane	C_12_H_34_O_3_Si_4_; 338.75 g/mol	26.430	21,459
58	1,4‐Bis(trimethylsilyl)benzene	C_12_H_22_Si_2_; 222.48 g/mol	26.430	21,459
59	Ethyl homovanillate, TMS derivative	C_13_H_20_O_4_Si; 268.38 g/mol	26.430	21,459
60	1,2‐Bis(trimethylsilyl)benzene	C_12_H_22_Si_2_; 222.48 g/mol	26.430	21,459
61	3‐Ethoxy‐1,1,1,5,5,5‐hexamethyl‐3‐(trimethylsilyl)trisiloxane	C_11_H_32_O_3_Si_4_; 324.73 g/mol	27.590	29,787
62	Trisiloxane, 1,1,1,5,5,5‐hexamethyl‐3,3‐bis[(trimethylsilyl)oxy]	C_12_H_36_O_4_Si_5_; 384.86 g/mol	27.590	29,787
63	Silane, trimethyl[5‐methyl‐2‐(1‐methylethyl)phenyl]	C_13_H_24_Si; 208.41 g/mol	27.590	29,787
64	1‐Isopropoxy‐5‐propyl‐2,3‐bis(trimethylsilyl)benzene	C_18_H_35_OSi_2_; 323.64 g/mol	27.590	29,787
65	3‐Isopropoxy‐1,1,1,5,5,5‐hexamethyl‐3‐(trimethylsilyl)trisiloxane	C_12_H_34_O_3_Si_4_; 338.75 g/mol	29.023	13,013
66	Methyltris(trimethylsiloxy)silane	C_10_H_30_O_3_Si_4_; 310.70 g/mol	29.023	13,013
67	4‐Hydroxyphenyllactic acid, ethyl ester, di‐TM	C_17_H_28_O_4_Si_2_; 352.57 g/mol	30.715	20,582
68	Tetrasiloxane, decamethyl‐	C_10_H_30_O_3_Si_4_; 310.70 g/mol	30.715	20,582
69	Benzoic acid, 4‐methyl‐2‐trimethylsilyloxy‐, trimethylsilyl ester	C_14_H_24_O_3_Si_2_; 296.51 g/mol	30.715	20,582
70	Hexasiloxane, 1,1,3,3,5,5,7,7,9,9,11,11‐dodeca‐	C_12_H_36_O_5_Si_6_; 428.95 g/mol	32.625	18,997
71	7,7,9,9,11,11‐Hexamethyl‐3,6,8,10,12,15‐hexaoxaheptadecane	C_17_H_36_O_6_; 336.46 g/mol	32.625	18,997
72	(R)‐(‐)‐Phenylephrine, bis(trimethylsilyl) ether	C_15_H_29_NO_2_Si_2_; 311.57 g/mol	42.870	22,043
73	Silicic acid, diethyl bis(trimethylsilyl) ester	C_10_H_28_O_4_Si_3_; 296.59 g/mol	42.870	22,043
74	3‐Methylsalicylic acid, 2TMS derivative	C_14_H_24_O_3_Si_2_; 296.51 g/mol	42.870	22,043
75	Octasiloxane, 1,1,3,3,5,5,7,7,9,9,11,11,13,13,15,15‐octamethyl‐	C_16_H_48_O_7_Si_8_; 577.26 g/mol	46.443	25,366
76	Methyltris(trimethylsiloxy)silane	C_10_H_30_O_3_Si_4_; 310.70 g/mol	48.860	25,811
77	Tetrasiloxane, decamethyl‐	C_10_H_30_O_3_Si_4_; 310.70 g/mol	48.860	25,811

The crude methanol extract of *Annona reticulata* Linn. leaves was found to contain 43 distinct components, according to the library search reports, which are represented in Table 3.

**TABLE 3:** Constituents detected by GC–MS analysis in *Annona reticulata* Linn.’s crude methanol extract.

Serial no.	Compound name	Molecular formula and molecular weight	Retention time	Area
1	Hexadecanoic acid, methyl ester	C_17_H_34_O_2_; 270.45 g/mol	13.822	506,819
2	Tridecanoic acid, 12‐methyl‐, methyl ester	C_15_H_30_O_2_; 242.40 g/mol	13.822	506,819
3	Tridecanoic acid methyl ester	C_14_H_28_O_2_; 228.37 g/mol	13.822	506,819
4	Decanoic acid, methyl ester	C_11_H_22_O_2_; 186.29 g/mol	13.822	506,819
5	Methyl tetradecanoate	C_15_H_30_O_2_; 242.39 g/mol	13.822	506,819
6	9,12‐Octadecadienal	C_18_H_32_O; 264.44 g/mol	15.758	43,473
7	Dichloroacetic acid, dodec‐9‐ynyl	C_14_H_18_Cl_2_O_2_; 289.18 g/mol	15.758	43,473
8	E,E‐1,9,17‐docasatriene	C_22_H_40_; 304.54 g/mol	15.758	43,473
9	9‐Dodecyn‐1‐ol	C_12_H_22_O; 182.30 g/mol	15.758	43,473
10	3‐Tetradecyne	C_14_H_28_; 196.36 g/mol	15.758	43,473
11	10‐Undecyn‐1‐ol	C_11_H_22_O; 170.29 g/mol	15.758	43,473
12	13‐Tetradece‐11‐yn‐1‐ol	C_14_H_28_O; 212.36 g/mol	15.758	43,473
13	E‐1,6‐Undecadiene	C_11_H_20_; 152.27 g/mol	15.758	43,473
14	11,14,17‐Eicosatrienoic acid, methyl ester	C_21_H_34_O_2_; 318.48 g/mol	15.653	557,717
15	cis,cis,cis‐7,10,13‐Hexadecatrienal	C_16_H_26_O; 234.37 g/mol	15.653	557,717
16	5‐Pentadecen‐7‐yne, (Z)‐	C_15_H_28_; 208.37 g/mol	15.653	557,717
17	3‐Heptadecen‐5‐yne, (Z)‐	C_17_H_32_; 236.43 g/mol	15.653	557,717
18	1‐Tetradecen‐3‐yne	C_14_H_26_; 194.35 g/mol	15.653	557,717
19	Phytol	C_20_H_40_O; 296.52 g/mol	15.760	257,362
20	3,4‐Dimethylcyclohexanol	C_8_H_16_O; 128.21 g/mol	15.760	257,362
21	1,7‐Octadien‐3‐ol, 2,6‐dimethyl‐	C_10_H_18_O; 154.24 g/mol	15.760	257,362
22	Oxirane, decyl‐	C_12_H_24_O; 184.31 g/mol	15.760	257,362
23	Methyl stearate	C_19_H_38_O_2_; 298.49 g/mol	15.900	278,138
24	Eicosanoic acid, methyl ester	C_21_H_42_O_2_; 326.55 g/mol	15.900	278,138
25	Octadecanoic acid, 17‐methyl‐, methyl ester	C_20_H_40_O_2_; 312.52 g/mol	15.900	278,138
26	Methyl tetradecanoate	C_15_H_30_O_2_; 242.39 g/mol	15.900	278,138
27	13‐Docosenamide, (Z)‐	C_22_H_41_NO; 335.56 g/mol	25.162	133,359
28	9‐Octadecenamide, (Z)‐	C_18_H_35_NO; 281.47 g/mol	25.162	133,359
29	8‐Methyl‐6‐nonenamide	C_10_H_21_NO; 171.28 g/mol	25.162	133,359
30	3‐Isopropoxy‐1,1,1,5,5,5‐hexamethyl‐3‐(trimethyl)pentane	C_13_H_30_O; 202.39 g/mol	29.528	13,558
31	Thymol, TMS derivative	C_13_H_21_OSi; 221.39 g/mol	29.528	13,558
32	1,2‐Bis(trimethylsilyl)benzene	C_12_H_22_Si_2_; 222.42 g/mol	29.528	13,558
33	1‐Isopropoxy‐5‐propyl‐2,3‐bis(trimethylsilyl)benzene	C_18_H_34_OSi_2_; 322.62 g/mol	29.528	13,558
34	2‐(N,N′,N′‐Trimethylhydrazino)‐1,3‐benzothiazole	C_10_H_14_N_3_S; 208.31 g/mol	29.528	13,558
35	1‐hexadecanesulfonamide, N‐(3‐aminopropyl)‐	C_19_H_41_N_2_O_2_S; 361.61 g/mol	29.528	13,558
36	3,5‐bis(trimethylsilyl)cyclohepta‐2,4,6‐trien‐1‐one	C_13_H_24_OSi_2_; 252.50 g/mol	29.528	13,558
37	Trisiloxane, 1,1,1,5,5,5‐hexamethyl‐3,3‐bis(trimethylsilyl)oxy‐	C_12_H_36_O_5_Si_5_; 400.86 g/mol	29.528	13,558
38	Trimethylsilyl‐di(timethylsiloxy)‐silane	C_9_H_27_O_2_Si_4_; 279.67 g/mol	29.528	13,558
39	Pterin‐6‐carboxylic acid	C_7_H_5_N_5_O_3_; 207.16 g/mol	32.298	26,957
40	1,4‐Bis(trimethylsilyl)benzene	C_12_H_22_Si_2_; 222.48 g/mol	32.298	26,957
41	Cholesterol 3‐O‐[[2‐acetoxy]ethyl]‐	C_31_H_52_O_2_; 455.72 g/mol	32.298	26,957
42	Androstane‐11,17‐dione, 3‐[(trimethylsilyl)oxy	C_22_H_34_O_2_Si; 358.58 g/mol	32.298	26,957
43	1,5,6,7‐Tetramethylbicyclo[3.2.0]hept‐6‐en‐3‐yl	C_11_H_19_; 151.26 g/mol	32.298	26,957

The compounds detected in GC–MS analysis are tentative identifications. However, certain detected peaks, particularly siloxane derivatives and silylated compounds, may originate from column bleed, derivatization artifacts, or instrumental contaminants. Therefore, further confirmation with optimized analytical conditions is required. Among the compounds detected from aqueous extract, L‐alanine, TMS derivative; 2‐(methylamino)ethanol, O‐trimethylsilyl; 3‐ethoxy‐1,1,1,5,5,5‐hexamethyl‐3‐(trimethylsilyl)trisiloxane; trisiloxane, 1,1,1,5,5,5‐hexamethyl‐3,3‐bis(trimethylsiloxy); heptasiloxane; 2,4,6‐cycloheptatrien‐1‐one, 3,5‐bis‐trimethylsilyl; hexestrol, 2TMS derivative; 3‐butoxy‐1,1,1,5,5,5‐hexamethyl‐3‐(trimethylsilyl)trisiloxane; methyltris(trimethylsiloxy)silane; 1‐isopropoxy‐5‐propyl‐2,3‐bis(trimethylsilyl)benzene; thymol, TMS derivative; silane, trimethyl[5‐methyl‐2‐(1‐methylethyl)phenyl]; trimethylsilyl‐di(trimethylsiloxy)‐silane; 3‐isopropoxy‐1,1,1,5,5,5‐hexamethyl‐3‐(trimethylsilyl)trisiloxane; 1,4‐bis(trimethylsilyl)benzene; ethyl homovanillate, TMS derivative; 1,2‐bis(trimethylsilyl)benzene; 3‐ethoxy‐1,1,1,5,5,5‐hexamethyl‐3‐(trimethylsilyl)trisiloxane; trisiloxane, 1,1,1,5,5,5‐hexamethyl‐3,3‐bis[(trimethylsilyl)oxy]; silane, trimethyl[5‐methyl‐2‐(1‐methylethyl)phenyl]; 1‐isopropoxy‐5‐propyl‐2,3‐bis(trimethylsilyl)benzene; 3‐isopropoxy‐1,1,1,5,5,5‐hexamethyl‐3‐(trimethylsilyl)trisiloxane; methyltris(trimethylsiloxy)silane; tetrasiloxane, decamethyl‐; benzoic acid, 4‐methyl‐2‐trimethylsilyloxy‐, trimethylsilyl ester; hexasiloxane, 1,1,3,3,5,5,7,7,9,9,11,11‐dodeca‐; (R)‐(‐)‐phenylephrine, bis(trimethylsilyl) ether; silicic acid, diethyl bis(trimethylsilyl) ester; 3‐methylsalicylic acid, 2TMS derivative; octasiloxane, 1,1,3,3,5,5,7,7,9,9,11,11,13,13,15,15‐octamethyl‐; methyltris(trimethylsiloxy)silane; and tetrasiloxane, decamethyl‐, the listed compounds might generate from column bleed or derivatization artifacts. In addition, among the components identified from methanol extract, thymol, TMS derivative; 1,2‐bis(trimethylsilyl)benzene; 1‐isopropoxy‐5‐propyl‐2,3‐bis(trimethylsilyl)benzene; 3,5‐bis(trimethylsilyl)cyclohepta‐2,4,6‐trien‐1‐one; trisiloxane, 1,1,1,5,5,5‐hexamethyl‐3,3‐bis(trimethylsilyl)oxy‐; trimethylsilyl‐di(timethylsiloxy)‐silane; 1,4‐bis(trimethylsilyl)benzene; and androstane‐11,17‐dione, 3‐[(trimethylsilyl)oxy, these enlisted components may create from instrumental contaminants. Furthermore, among the detected 77 compounds of aqueous extract, octadecanoic acid, 17‐methyl‐methyl ester, has been reported to contain antimicrobial and antiviral activity [55, 56]; 13‐docosenamide (z)‐ has been reported to possess good antibacterial activity [57, 58]; thiophene derivatives and thymol derivative reported to have antimicrobial activity [59, 60]; cyclobutanol is reported to possess antifungal activity [61]; and 1,3‐dioxolane‐4‐methanol contains both antibacterial and antifungal activity, as per report [62], whereas among the 43 compounds of methanol extract, hexadecanoic acid, methyl ester; tridecanoic acid methyl ester (TAME); Cis,cis,cis‐7,10,13‐hexadecatrienal; and eicosanoic acid, methyl ester, have been reported to possess antibacterial properties [63–66]; 3‐tetradecyne, 5‐pentadecen‐7‐yne‐z‐, phytol, thymol derivative, 1,2‐bis (trimethylsilyl) benzene, and cholesterol derivatives are reported to have broad‐spectrum antibacterial property [67–71]; 13‐docosenamide (z)‐ is reported to contain both antibacterial and antifungal potentials [57]; methyl stearate is reported to possess antifungal property [72]; and 8‐methyl‐6‐nonenamide has been reported to contain strong antiviral and antibacterial potentials [73]. Additionally, as per reports, among these listed compounds, 1,3‐dioxolane‐4‐methanol and cyclopropyl in aqueous extract possess good insecticidal properties [74, 75], whereas 10‐undecyn‐1‐ol in methanol extract contains insecticidal activities [76]. Moreover, for both extracts, TAME may possess antidiarrheal properties, according to a previous report [64]. Moreover, the biological activities related to some identified compounds are based on literature reports and should be considered hypothetical, as no isolation or confirmatory structural characterization was performed in the present study. Further purification and spectroscopic validation would be required to definitively correlate these compounds with the observed bioactivities and to confirm the suspected bioactivities.

### 3.3. In Vitro Evaluation of Antibacterial Properties

Utilizing the disc diffusion method, the antibacterial potentials of the test samples were investigated. As negative control, DMSO yielded negligible outcomes. Methanol and aqueous leaf extracts prevented the growth of both gram‐positive and gram‐negative bacteria; however, the methanolic fraction demonstrated stronger antibacterial potentials than the aqueous fraction. Although tetracycline showed the strongest antibacterial properties against all bacterial strains in this investigation, it also showed the highest activity against *L. monocytogenes*, indicating its broad‐spectrum efficacy. Methanol leaf extract at 250 mg/mL dose demonstrated significant antibacterial activity against all bacterial strains; highest activity was obtained against *M. tuberculosis*, while the aqueous leaf extract also exhibited a certain level of antibacterial activity at a 250 mg/mL dose, with the greatest activity against *K. pneumoniae* (Table 4). Therefore, the antibacterial activities were declined in the following order:

(1) standard tetracycline>MEAR 250250>AEAR .

**TABLE 4:** Zone of inhibition (mm) of aqueous and methanol fractions from *Annona reticulata* Linn. leaves and tetracycline (standard) against some bacteria.

Organisms	Zone of inhibition (mm) (mean ± SD)			
	Standard	AEAR 250	MEAR 250	DMSO
*B. subtilis*	18.7 ± 0.5	8.3 ± 0.5	14.7 ± 0.5	0
*S. aureus*	18.3 ± 0.5	10.7 ± 0.5	17.7 ± 0.5	0
*M. tuberculosis*	19.7 ± 0.5	10.3 ± 0.5	19.3 ± 0.5	0
*L. monocytogenes*	20.7 ± 0.5	11.3 ± 0.5	18.3 ± 0.5	0
*E. coli*	20.3 ± 0.5	8.7 ± 0.5	16.3 ± 0.5	0
*V. cholerae*	18.3 ± 0.5	9.7 ± 0.5	17.3 ± 0.5	0
*K. pneumoniae*	19.3 ± 0.5	11.7 ± 0.5	16.7 ± 0.5	0

*Note:* Here, AEAR means aqueous extract of *Annona reticulata* Linn. leaves. MEAR means methanol extract of *Annona reticulata* Linn. leaves.

However, antibacterial activity observed with the test crude plant extract was evaluated at high concentrations (250 mg/mL), which may influence inhibition zones due to diffusion effects. Therefore, direct comparison with standard antibiotics (e.g., tetracycline) was interpreted cautiously.

MIC is the lowest concentration of an antimicrobial medication that, after an overnight incubation time, will stop a microorganism from growing visibly. The aqueous extract exhibited the highest activities against *S. aureus* and *M. tuberculosis* with an MIC value of 31.25 mg/mL, whereas the methanolic leaf extract was more toxic to all the bacterial strains, with observed lower MIC values. The greatest activity was obtained for the methanolic leaf extract against *S. aureus*, *M. tuberculosis*, and *V. cholerae*, with an MIC value of 15.625 mg/mL (Table 5). Moreover, both methanol and aqueous leaf extracts were effective against all test bacterial strains and good antibacterial activity was obtained for both extracts.

**TABLE 5:** MIC of aqueous and methanol extracts of *Annona reticulata* Linn. leaves.

Organisms	Minimum inhibitory concentration (MIC) (mg/mL)	
	AEAR	MEAR
*B. subtilis*	62.5	31.25
*S. aureus*	31.25	15.625
*M. tuberculosis*	31.25	15.625
*L. monocytogenes*	62.5	31.25
*E. coli*	125	62.5
*V. cholerae*	62.5	15.625
*K. pneumoniae*	62.5	31.25

*Note:* Here, AEAR means aqueous extract of *Annona reticulata* Linn. leaves. MEAR means methanol extract of *Annona reticulata* Linn. leaves.

An assay used in microbiology to assess bacterial drug susceptibility is the disc diffusion test. It is used in drug research and diagnostic labs [77]. In this investigation, the methanolic leaf fraction showed significant antibacterial properties, while the aqueous leaf extract demonstrated moderate to good antibacterial activities. However, it should be noted that, unlike purified antibiotics, crude extracts contain diverse phytochemicals that may influence diffusion behavior and apparent inhibition zones in disc diffusion assays. Data from phytochemical screening indicate the existence of tannins, flavonoids, glucosides, alkaloids, steroids, glycosides, and carbohydrates (monosaccharides and reducing sugars) in aqueous and methanolic leaf fractions. As per reports, all these compounds contain antibacterial properties [34–42]. Through the destruction of the bacterial cell membrane, the inhibition of vital enzymes and processes such as the synthesis of proteins and cell walls, the disruption of bacterial metabolic pathways and virulence factors, including the suppression of biofilm formation, and interference with DNA replication, phytochemicals provide antibacterial activity. Additionally, some phytochemicals can work with traditional antibiotics, producing synergistic activity to block bacterial efflux pumps, which cause antibiotic resistance. This might result in the creation of novel antibacterial therapies [78, 79]. In addition, GC–MS analytical data indicated that both aqueous and methanol extracts contained few components with probable antimicrobial properties. For aqueous extract, such compounds include octadecanoic acid,17‐methyl‐methyl ester, which is reported to have antimicrobial and antiviral activity [55, 56]; 13‐docosenamide (z)‐, which is reported to possess good antibacterial activity [57, 58]; thiophene derivatives and thymol derivative, which have been reported to contain antimicrobial activity [59, 60]; cyclobutanol, which is reported to possess antifungal activity [61]; and 1,3‐dioxolane‐4‐methanol, which is reported to have both antibacterial and antifungal activity [62]. For methanol extract, such components include hexadecanoic acid, methyl ester; TAME; cis,cis,cis‐7,10,13‐hexadecatrienal; and eicosanoic acid, methyl ester, which have been reported to have antibacterial properties [63–66]; 3‐tetradecyne; 5‐pentadecen‐7‐yne‐z‐; phytol; thymol derivative; cholesterol derivatives that are reported to possess broad‐spectrum antibacterial properties [67–71]; 13 docosenamide (z)‐, which is reported to have both antibacterial and antifungal potentials [57]; methyl stearate that is reported to contain antifungal property [72]; and 8‐methyl‐6‐nonenamide, which is reported to have strong antiviral and antibacterial potentials [73]. Many researchers have assessed the antibacterial activities of various *Annona reticulata* Linn. extracts because the plant has a variety of antimicrobial components [15, 80, 81]. Besides that, the antimicrobial properties of a number of different *Annonaceae* family plants, including *Annona mucosa* and *Annona muricata*, have also been investigated [82–84]. The outcomes of those investigations were comparable to the outcomes obtained from *Annona reticulata* Linn. in our study. These findings provide preliminary evidence of antibacterial activities; however, further investigations are required to identify and confirm the compounds responsible for these bioactivities.

### 3.4. In Vivo Assessment of Insecticidal Activity

The insecticidal activities of methanol and aqueous leaf extracts were evaluated against *T. castaneum* and *P. americana*. The mortality rate for methanol‐treated insects (control) was zero despite an increase in concentration. Insects that were exposed to standard permethrin demonstrated high mortality percentages, starting from 25%–20% to 100% against *T. castaneum* and *P. americana*, indicating the notable insecticidal properties of permethrin. Methanolic leaf extract from *Annona reticulata* Linn. exhibited the highest insecticidal activity in this study against both insects. The percentage of mortality obtained from the methanolic leaf extract against *T. castaneum* began with 30% for 1 mg/2mL, which was even higher than the outcome of permethrin at this initial stage. The mortality percentage gradually reached to 100% for methanol extract against *T. castaneum*, whereas against *P. americana* at 1 mg/2 mL concentration, the obtained percentage of mortality was 20%, which was equal to the effect observed from permethrin; the mortality rate increased to 100% with an increase in concentration of extract. On the other hand, aqueous leaf extract also demonstrated good insecticidal activity, but the activity was lower than that of the methanol extract. Against *T. castaneum* and *P. americana*, aqueous extract exhibited mortality percentage of 20% and 10% at 1 mg/mL concentration, respectively, which consequently reached to 90% against both insects (Table 6). In the following declining series, the percentage of mortality was noted:

(2) MEAR>standard>AEAR.

**TABLE 6:** Percentage mortality of insects in different concentrations of aqueous and methanol leaf extracts of *Annona reticulata* Linn.

Concentration (mg/2 mL)	Against *T. castaneum*				Against *P. americana*			
	AEAR (%)	MEAR (%)	Standard (%)	Control (%)	AEAR (%)	MEAR (%)	Standard (%)	Control (%)
1	20	30	25	0	10	20	20	0
5	40	50	45	0	30	50	40	0
10	55	65	60	0	50	70	60	0
50	70	80	80	0	70	90	80	0
100	90	100	100	0	90	100	100	0

*Note:* Here, AEAR means aqueous extract of *Annona reticulata* Linn. leaves. MEAR means methanol extract of *Annona reticulata* Linn. leaves.

**FIGURE 3:**
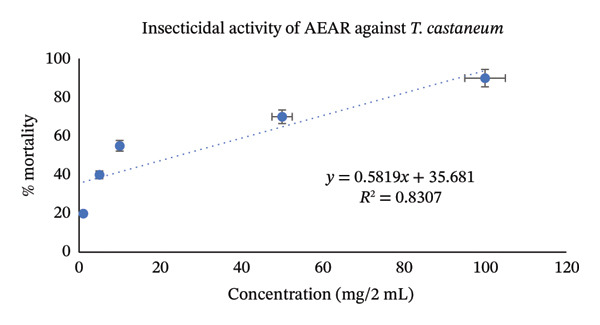
Graphical representation of insecticidal potential of aqueous leaf extract of *Annona reticulata* Linn. against *T. castaneum*.

**FIGURE 4:**
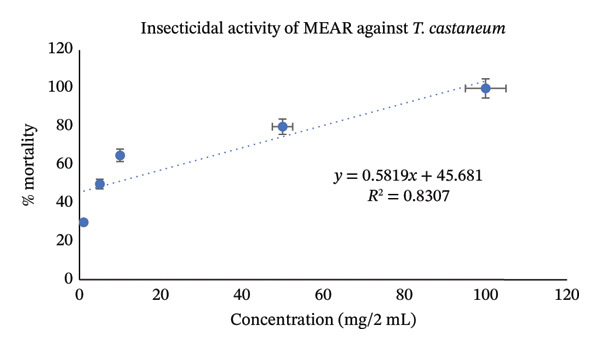
Graphical representation of insecticidal potential of methanolic leaf extract of *Annona reticulata* Linn. against *T. castaneum*.

**FIGURE 5:**
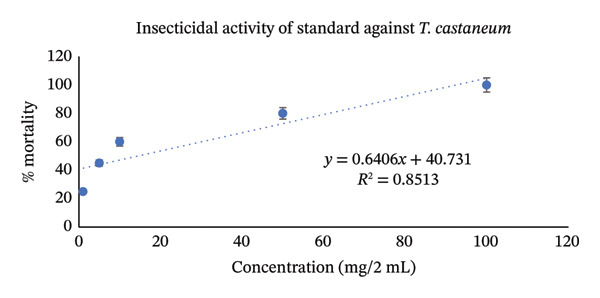
Graphical representation of insecticidal potential of standard (permethrin) against *T. castaneum*.

**FIGURE 6:**
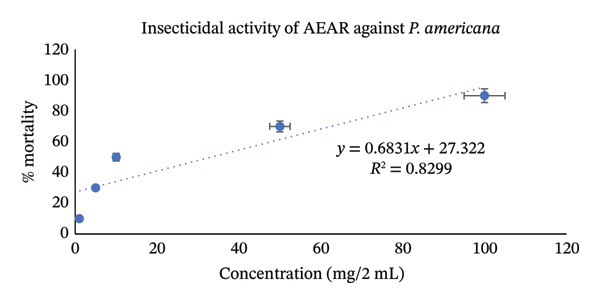
Graphical representation of insecticidal potential of aqueous leaf extract of *Annona reticulata* Linn. against *P. americana*.

**FIGURE 7:**
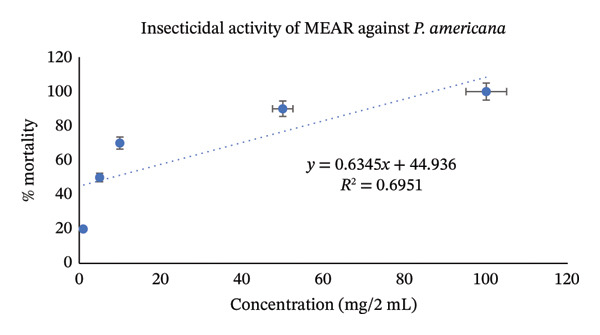
Graphical representation of insecticidal potential of methanolic leaf extract of *Annona reticulata* Linn. against *P. americana*.

**FIGURE 8:**
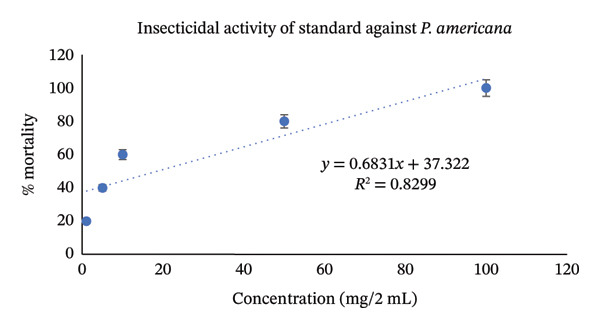
Graphical representation of insecticidal potential of standard (permethrin) against *P. americana*.

Utilizing the dose–response relationships, median lethal dose (LD_50_) values were calculated for standard, aqueous, and methanol leaf extracts; these values are represented in Table 7. LD_50_ represents the dose that causes death in 50% of test animals. Methanol leaf extract was found to be very toxic to both *T. castaneum* and *P. americana*, with LD_50_ values of 7.42 mg/2mL (95% CI: 6.50–8.35 mg/2 mL) and 7.98 mg/2mL (95% CI: 7.10–8.90 mg/2 mL). These values were even more significant than the values obtained from standard permethrin. However, aqueous leaf extract also demonstrated good insecticidal activities against both *T. castaneum* and *P. americana* with LD_50_ values of 24.61 mg/2 mL (95% CI: 22.30–27.10 mg/2 mL) and 33.19 mg/2 mL(95% CI: 30.10–36.50 mg/2 mL) (Figures 3, 4, 5, 6, 7, and 8). Here, 95% confidence intervals were calculated to indicate the precision of the estimates and enhance transparency.

**TABLE 7:** LD_50_ values of standard, aqueous, and methanol leaf extracts of *Annona reticulata* Linn.

Sample	LD_50_ (mg/2 mL)	
	Against *T. castaneum*	Against *P. americana*
AEAR	24.61	33.19
MEAR	7.42	7.98
Standard	14.47	18.56

Data obtained from the insecticidal study demonstrated that the insecticidal properties of both extracts increased with increasing concentration, so the insecticidal activities of methanolic and aqueous extracts are dose‐dependent. However, the methanolic extract exhibited lower LD_50_ values than the aqueous extract and also lower than the standard insecticide permethrin, which indicates significant insecticidal potential of the crude plant extract. While comparing crude plant extracts with the synthetic insecticide permethrin, such comparisons are indicative rather than representative of practical efficacy. As the synthetic insecticides are very harmful to the environment and living beings, searching for such natural alternatives can be an ideal approach. Although the extracts demonstrated notable insecticidal activities under laboratory conditions, the concentrations required to achieve these effects were relatively high and may not directly reflect practical field applicability. In practical conditions, factors such as stability of the extract, method of application, and environmental degradation may influence the actual performance of the crude extracts. Therefore, the present findings should be considered as preliminary evidence of insecticidal potential. Further studies involving formulation development, lower effective doses, and field‐based evaluations are necessary to determine the ecological relevance and practical usability of these plant extracts as natural insecticidal agents. Furthermore, phytochemical screening test showed that both aqueous and methanol extracts contained carbohydrate, glycoside, alkaloid, flavonoid, tannin, steroid, and glucoside—all of which have insecticidal properties as per reports [43–49]. Insects can be killed or deterred by phytochemicals through repellent properties, inhibition of vital enzymes like proteases, disruption of insect cell activities, or interference with the neurological, digestive, or endocrine systems. Terpenoids, alkaloids, phenols, and flavonoids are examples of specific phytochemical classes that target various insect processes by interfering with feeding, serving as antifeedants, or even directly killing insects [85, 86]. According to the GC–MS analytical report, the aqueous extract included 1,3‐dioxolane‐4‐methanol and cyclopropyl carbinol, both of which are reported to have potent insecticidal effects [74, 75], while 10‐undecyn‐1‐ol, a compound that is reported to possess significant insecticidal action, was found in the methanolic fraction [76]. The mosquito‐larvicidal ability of several extracts from *Annona reticulata* Linn. has been evaluated and confirmed by numerous researchers [87, 88]. The insecticidal potentials of silver nanoparticles derived from *Annona reticulata* Linn. were also evaluated [89]. The outcomes of our investigation are consistent with theirs, indicating the significant insecticidal properties of *Annona reticulata* Linn; however, further mechanistic studies are required to confirm the current findings.

### 3.5. *In Vivo* Evaluation of Antidiarrheal Potential

#### 3.5.1. Castor Oil–Induced Diarrhea

Among the obtained onset of diarrhea values, the negative control group demonstrated the lowest value and the standard group treated with loperamide showed the highest value. Both methanol and aqueous leaf extracts prolonged the onset of diarrhea values in a significant amount, and the effects increased with increasing dosage of the extracts. Additionally, the negative control group exhibited the highest number of feces values, which was lowered notably by loperamide. Both aqueous and methanol leaf extracts lowered the total number of feces values significantly at both 200 and 400 mg/kg of body weight dosages, with percentage of defecation inhibition values of 30.14% and 31.51% at 400 mg/kg of body weight dosage. Methanolic and aqueous leaf extracts also demonstrated significant outcomes at reducing the amount of wet diarrheal feces at both 200 and 400 mg/kg doses, with percentage inhibition of diarrhea values of 41.86% and 44.19% at 400 mg/kg dosages; these outcomes were even higher than those obtained from the standard drug loperamide at 5 mg/kg dosage (Table 8). The antidiarrheal activities decreased in the following order:

(3) MEAR 400400200200>AEAR >Standard>MEAR >AEAR .

**TABLE 8:** Effect of aqueous and methanol leaf extracts of *Annona reticulata* Linn. on castor oil–induced diarrhea in rats.

Group	Onset of diarrhea (mean ± SEM)	Total number of feces (mean ± SEM)	% inhibition of defecation	Total number of diarrheal feces (mean ± SEM)	% inhibition of diarrhea
Negative Control	14.50 ± 0.65	18.25 ± 0.48	—	10.75 ± 0.65	—
Standard	90.25 ± 2.66^∗∗∗^	13.00 ± 0.41^∗∗∗^	28.77%	6.50 ± 0.48^∗∗∗^	39.53%
AEAR 200	57.50 ± 1.85^∗∗∗^	13.75 ± 0.65^∗∗∗^	24.66%	7.25 ± 0.66^∗∗∗^	32.56%
AEAR 400	76.75 ± 2.29^∗∗∗^	12.75 ± 0.29^∗∗∗^	30.14%	6.25 ± 0.48^∗∗∗^	41.86%
MEAR 200	60.50 ± 1.32^∗∗∗^	13.50 ± 0.48^∗∗∗^	26.03%	6.75 ± 0.29^∗∗∗^	37.21%
MEAR 400	82.50 ± 1.56^∗∗∗^	12.50 ± 0.25^∗∗∗^	31.51%	6.00 ± 0.41^∗∗∗^	44.19%

*Note:* Standard is loperamide hydrochloride. AEAR means aqueous extract of *Annona reticulata* Linn. leaves. MEAR means methanol extract of *Annona reticulata* Linn. leaves.

^∗^ *p* < 0.05 (Significance).

^∗∗^ *p* < 0.01 (Highly significance).

^∗∗∗^ *p* < 0.001 (Very highly significance) against control.

#### 3.5.2. Castor Oil–Induced Enteropooling

Intestinal weight and volume were notably and dose‐dependently decreased by both methanol and aqueous leaf extracts at the 200 and 400 mg/kg doses (Table 9). At dosages of 200 and 400 mg/kg, the intestinal volume was reduced by 53.65% and 41.09% with the methanolic extract, and by 51.59% and 39.27% with the aqueous extract, respectively. Additionally, the standard drug loperamide (5 mg/kg) considerably reduced the buildup of intestinal fluid (48.63%, *p* < 0.001).

**TABLE 9:** Effect of aqueous and methanol leaf extracts on castor oil–induced enteropooling in rats.

Group	Weight of intestinal content (g) (mean ± SEM)	Volume of intestinal content (mL) (mean ± SEM)	Inhibition (%)
Negative control	4.38 ± 0.06	2.92 ± 0.07	—
Standard	2.25 ± 0.25^∗∗∗^	1.78 ± 0.32^∗∗∗^	48.63%
AEAR 200	2.66 ± 0.48^∗∗∗^	1.96 ± 0.08^∗∗∗^	39.27%
AEAR 400	2.12 ± 0.03^∗∗∗^	1.67 ± 0.06^∗∗∗^	51.59%
MEAR 200	2.58 ± 0.29^∗∗∗^	1.91 ± 0.29^∗∗∗^	41.09%
MEAR 400	2.03 ± 0.08^∗∗∗^	1.66 ± 0.03^∗∗∗^	53.65%

*Note:* Standard is loperamide hydrochloride. AEAR means aqueous extract of *Annona reticulata* Linn. leaves. MEAR means methanol extract of *Annona reticulata* Linn. leaves.

^∗^ *p* < 0.05 (significant).

^∗∗^ *p* < 0.01 (highly significant).

^∗∗∗^ *p* < 0.001 (very highly significant) against control.

#### 3.5.3. Gastrointestinal Motility Test

In comparison with the control group, methanolic and aqueous leaf fractions of *Annona reticulata* Linn. greatly decreased the amount of gastrointestinal distance that the charcoal meal went through the rats’ bodies. At 400 mg/kg body weight dosage, methanol and aqueous leaf extracts exhibited 52.78% and 50.89% inhibition in the passage of charcoal meal. In addition, a notable reduction in the propulsion of charcoal meal (48.81%) through the GIT was caused by loperamide (5 mg/kg) (Table 10).

**TABLE 10:** Effect of aqueous and methanol leaf extracts on small intestinal transition in rats.

Group	Total length of intestine (cm) (mean ± SEM)	Distance traveled by marker (cm) (mean ± SEM)	Inhibition (%)
Negative control	105.7 ± 2.24	100.8 ± 3.07	—
Standard	102.5 ± 1.68	51.6 ± 2.55^∗∗∗^	48.81%
AEAR 200	99.3 ± 3.06	68.4 ± 2.34^∗∗∗^	32.14%
AEAR 400	101.8 ± 2.44	49.5 ± 1.76^∗∗∗^	50.89%
MEAR 200	103.6 ± 3.13	62.7 ± 1.82^∗∗∗^	37.79%
MEAR 400	98.5 ± 2.12	47.6 ± 3.08^∗∗∗^	52.78%

*Note:* Standard is loperamide hydrochloride. AEAR means aqueous extract of *Annona reticulata* Linn. leaves. MEAR means methanol extract of *Annona reticulata* Linn. leaves.

^∗^ *p* < 0.05 (significant).

^∗∗^ *p* < 0.01 (highly significant).

^∗∗∗^ *p* < 0.001 (very highly significant) against control.

Castor oil–induced diarrhea in mice serves as a model for assessing the efficacy of treatments on diarrhea. Castor oil–induced diarrhea is caused by an excess of fluid in the intestines, which is a result of inflammation and damage to the intestinal mucosa [90]. Nonetheless, it is well recognized that the most potent component of castor oil, ricinoleic acid, induces diarrhea by means of a hypersecretory response [91]. The study’s findings proved that *Annona reticulata* Linn.’s aqueous and methanol leaf fractions worked well to treat diarrhea. Carbohydrate, glycoside, alkaloid, flavonoid, and tannin are reported to possess antidiarrheal properties; these compounds were identified in both extracts via phytochemical screening test [50–54]. The main ways that phytochemicals prevent diarrhea are by lowering intestinal motility and fluid secretion. Important phytochemicals such as flavonoids and tannins increase the absorption of water and electrolytes while blocking peristaltic motions. Other substances, such as terpenoids and alkaloids, can decrease inflammatory mediators like prostaglandins and change calcium currents to impede intestinal transit and disrupt intestinal fluid formation [92]. Furthermore, GC–MS analytical data state that both aqueous and methanol extracts contain TAME, which is reported to have antidiarrheal properties. As per reports, through its broad‐spectrum antibacterial properties, which disrupt cellular structure and cause leakage in pathogenic bacteria such as *Salmonella enterica* and *Enterococcus faecalis*, TAME reduces diarrhea. According to studies, TAME inhibits the growth and viability of bacteria that cause diarrheal infections by causing significant bacterial cell rupture. Also, the compound might make current antibiotics more effective, which could lessen their overuse [64]. Some researchers evaluated and confirmed that *Annona reticulata* Linn.’s stem bark extracts have antidiarrheal potentials [93]. In our study, the outcomes indicate notable antidiarrheal properties of aqueous and methanolic leaf extracts of *Annona reticulata* Linn.

This study was designed as an initial screening of the insecticidal, antibacterial, and antidiarrheal potentials of the methanol and aqueous extracts of *Annona reticulata* Linn. leaves. Since the primary goal was to identify potential bioactivities rather than to establish precise mechanisms of action, the study is exploratory in nature; for this reason, the statistical analyses were limited to *p*‐values, and detailed ANOVA parameters for multiple comparisons were not applied. Therefore, any observed effects or statistically significant differences should be interpreted as preliminary indications of activity, and mechanistic conclusions cannot be drawn at this stage. Further targeted studies are required to confirm these findings and elucidate the underlying mechanisms. Additionally, while the observed biological activities may be related to the phytoconstituents identified in the extracts, it should be noted that the compounds detected by GC–MS were only tentatively identified and have not been directly isolated and experimentally confirmed. Therefore, the correlations between the identified phytochemicals from GC–MS analysis and the observed insecticidal, antibacterial, and antidiarrheal activities are presented as preliminary and hypothetical; further targeted studies are required for confirmation of these bioactivities. Furthermore, a limitation of the antibacterial evaluation is that the test extracts were tested at a relatively high concentration (250 mg/mL) compared to the standard drug tetracycline. This high dose was used to ensure the detection of potential antibacterial activities during preliminary screening. However, the observed effects may not directly reflect activity at physiologically relevant concentrations. Therefore, further study incorporating a lower dose is required to determine the antibacterial activities at physiologically relevant concentrations. Although the current findings indicate potential therapeutic and insecticidal properties of *Annona reticulata* Linn. leaf extracts, further mechanistic studies, isolation, characterization of bioactive constituents, and comprehensive toxicity assessments are required to validate these potentials.

## 4. Conclusion

The outcomes of this investigation offer compelling proof that methanolic and aqueous extracts of leaves of *Annona reticulata* Linn. exhibited good antibacterial, insecticidal, and antidiarrheal potentials. At dosages of 200 and 400 mg/kg, the observed antidiarrheal activities were rapid, persistent, and statistically significant. The mechanisms of antidiarrheal activity may be clearly understood by determining the antidiarrheal effects in various animal models. To determine the chemicals responsible for these activities, GC–MS analysis and phytochemical screening results were utilized. To identify the specific processes behind the plant’s apparent pharmacological actions, more chemical and pharmacological research is necessary. However, separating the bioactive compounds from the extracts and testing them further could assist in clarifying the mechanisms of action by which various bioactivities were attained and open the door to drawing clear conclusions about the current outcomes.

## Funding

No funding was received for this manuscript.

## Conflicts of Interest

The authors declare no conflicts of interest.

## Data Availability

The data that support the findings of this study are available from the corresponding author upon reasonable request.

## References

[1] Suresh P. S. , Gupta S. S. , and Sharma U. , Insight into Coronaviruses and Natural Products-based Approach for COVID-19 Treatment, Studies in Natural Products Chemistry. (2022) 74, 443–469.

[2] Saini N. , Lather V. , and Gahlawat S. K. , Exploring Phytochemicals from Himalayan Medicinal Plants as Novel Therapeutic Agents, Anti-Cancer Agents in Medicinal Chemistry-Anti-Cancer Agents.(2022) 22, no. 9, 1674–1698, 10.2174/1871520621666211015141020. 34773963

[3] Eloff J. N. , Avoiding Pitfalls in Determining Antimicrobial Activity of Plant Extracts and Publishing the Results, BMC Complementary and Alternative Medicine. (2019) 19, no. 1, 10.1186/s12906-019-2519-3, 2-s2.0-85066471535. PMC6530048 31113428

[4] Cheng G. , Dai M. , Ahmed S. , Hao H. , Wang X. , and Yuan Z. , Antimicrobial Drugs in Fighting Against Antimicrobial Resistance, Frontiers in Microbiology. (2016) 7, 10.3389/fmicb.2016.00470, 2-s2.0-84966270200. PMC4824775 27092125

[5] Zazharskyi V. V. , Davydenko P. , Kulishenko O. , Borovik I. V. , and Brygadyrenko V. V. , Antimicrobial Activity of 50 Plant Extracts, Biosystems Diversity. (2019) 27, no. 2, 163–169, 10.15421/011922, 2-s2.0-85071276788.

[6] Vaou N. , Stavropoulou E. , Voidarou C. , Tsigalou C. , and Bezirtzoglou E. , Towards Advances in Medicinal Plant Antimicrobial Activity: a Review Study on Challenges and Future Perspectives, Microorganisms. (2021) 9, no. 10, 10.3390/microorganisms9102041. PMC8541629 34683362

[7] Fazeli-Dinan M. , Mostafavi A. , Osia-Laghab H. et al., Evaluation of Medicinal Plant Extracts as Safe Alternatives to Chemical Insecticides for Enhancing Food Safety and Public Health, Journal of health research in community.(2025) 11, no. 1, 50–62.

[8] Motazedian N. , Davoodi A. , Aleosfoor M. , and Bandani A. R. , Insecticidal Activity of Five Medicinal Plant Essential Oils Against the Cabbage Aphid, Brevicoryne brassicae, Journal of crop protection. (2025) 3, no. 2, 137–146.

[9] Mohammad M. Y. , Haniffa H. M. , Shakya A. K. , Naik R. R. , and Sivaranjan T. , Evaluation of Five Medicinal Plants for the Management of Sitophilus oryzae in Stored Rice and Identification of Insecticidal Compound, Heliyon. (2024) 10, no. 10, 10.1016/j.heliyon.2024.e30793. PMC11103461 38770290

[10] Mosisa G. B. , Melesie Taye G. , Abula T. , and Alemayehu Gadisa D. , Evaluation of Anti-diarrheal Activity of 80% Methanol Extracts of Vernonia amygdalina Delile (Asteraceae) Leaves in Mice, Journal of Experimental Pharmacology. (2020) 12, 455–462, 10.2147/JEP.S282669. 33177891 PMC7652236

[11] Rawat P. , Singh P. K. , and Kumar V. , Evidence Based Traditional Anti-diarrheal Medicinal Plants and Their Phytocompounds, Biomedicine & Pharmacotherapy. (2017) 96, 1453–1464, 10.1016/j.biopha.2017.11.147, 2-s2.0-85036631506. 29217158

[12] Rahman M. K. , Chowdhury M. A. , Islam M. T. , Chowdhury M. A. , Uddin M. E. , and Sumi C. D. , Evaluation of Antidiarrheal Activity of Methanolic Extract of Maranta arundinacea Linn. Leaves, Advances in Pharmacological and Pharmaceutical Sciences. (2015) 2015, no. 1, 1–6, 10.1155/2015/257057, 2-s2.0-84939825885. PMC4543376 26346095

[13] Grace D. , Food Fraud, Encyclopedia of Food Security and Sustainability. (2018) 238–248.

[14] Sharma H. and Ozogul F. , Mass Spectrometry-based Techniques for Identification of Compounds in Milk and Meat Matrix, Advances in Food and Nutrition Research. (2023) 104, 43–76, 10.1016/bs.afnr.2022.10.004. 37236734

[15] Jamkhande P. G. , Wattamwar A. S. , Kankudte A. D. , Tidke P. S. , and Kalaskar M. G. , Assessment of Annona reticulata Linn. Leaves Fractions for Invitro Antioxidative Effect and Antimicrobial Potential Against Standard Human Pathogenic Strains, Alexandria Journal of Medicine. (2016) 52, no. 1, 19–25, 10.1016/j.ajme.2014.12.007.

[16] Islam M. R. , Naima J. , Proma N. M. , Hussain M. S. , Uddin S. N. , and Hossain M. K. , In-vivo and in-vitro Evaluation of Pharmacological Activities of Ardisia solanacea Leaf Extract, Clinical Phytoscience. (2019) 5, no. 1, 10.1186/s40816-019-0128-9.

[17] Sangeetha V. S. , Babu M. , and Lawrence B. , Phytochemical Analysis of Annona reticulata L. Leaf Extracts, International Research Journal of Pharmaceutical and Applied Sciences. (2014) 4, no. 5, 4–8.

[18] Yadav M. , Chatterji S. , Gupta S. K. , and Watal G. , Preliminary Phytochemical Screening of Six Medicinal Plants Used in Traditional Medicine, International Journal Pharmacy Science. (2014) 6, no. 5, 539–542.

[19] Sheel R. , Nisha K. , and Kumar J. , Preliminary Phytochemical Screening of Methanolic Extract of Clerodendron Infortunatum, IOSR Journal of Applied Chemistry. (2014) 7, no. 1, 10–13, 10.9790/5736-07121013.

[20] Labiad M. H. , Harhar H. , Ghanimi A. , and Tabyaoui M. , Phytochemical Screening and Antioxidant Activity of Moroccan Thymus Satureioïdes Extracts, Journal of Materials and Environmental Sciences. (2017) 8, no. 6, 2132–2139.

[21] Ajuru M. G. , Williams L. F. , and Ajuru G. , Qualitative and Quantitative Phytochemical Screening of Some Plants Used in Ethnomedicine in the Niger Delta Region of Nigeria, Journal of Food and Nutrition Sciences. (2017) 5, no. 5, 198–205, 10.11648/j.jfns.20170505.16.

[22] Rani D. J. , Rahinidevi R. , and Vidyashri M. , Phytochemical Screening and Antimicrobial Activity of Various Solvent Extracts of Annona reticulata Leaves, International Journal of Science Inventions Today. (2013) 2, no. 5, 347–358.

[23] Kabbo T. B. , Rahman F. S. , Rana M. S. , and Dash P. R. , Evaluation of Antioxidant, Cytotoxic, and Anticancer Activities of Methanol Extract of Annona reticulata Linn. Leaves, Scientifica. (2025) 2025, no. 1, 10.1155/sci5/5166065. PMC12411056 40919384

[24] Dhiman A. , Nanda A. , Ahmad S. , and Narasimhan B. , In Vitro Antimicrobial Activity of Methanolic Leaf Extract of Psidium guajava L, Journal of Pharmacy and BioAllied Sciences. (2011) 3, no. 2, 226–229, 10.4103/0975-7406.80776, 2-s2.0-79957538332. 21687350 PMC3103916

[25] Umer S. , Tekewe A. , and Kebede N. , Antidiarrhoeal and Antimicrobial Activity of Calpurnia aurea Leaf Extract, BMC Complementary and Alternative Medicine. (2013) 13, no. 1, 10.1186/1472-6882-13-21, 2-s2.0-84872777451. PMC3565866 23351272

[26] Ahmad M. , Saeed F. , and Noor Jahan M. , Evaluation of Insecticidal and Antioxidant Activity of Selected Medicinal Plants, Journal of Pharmacognosy and Photochemistry. (2013) 2, no. 3, 153–158.

[27] Mares M. M. , Murshed M. , Aljawdah H. , Hailan W. A. , and Al-Quraishy S. , In Vitro Assessment of the Insecticidal Activity of Nerium oleander Extract Against German Cockroaches (Blattella germanica), Indian Journal of Animal Research. (2024) 58, no. 10, 1751–1757, 10.18805/ijar.bf-1727.

[28] Kabbo T. B. , Rana S. , and Dash P. R. , Assessment of in Vivo Analgesic, Anti‐Inflammatory and Wound Healing Properties of Aqueous Leaf Extract of Annona reticulata Linn, The Scientific World Journal. (2025) 2025, no. 1, 10.1155/tswj/4535663. PMC12623095 41256237

[29] Sisay M. , Engidawork E. , and Shibeshi W. , Evaluation of the Antidiarrheal Activity of the Leaf Extracts of Myrtus communis Linn (Myrtaceae) in Mice Model, BMC Complementary and Alternative Medicine. (2017) 17, no. 1, 10.1186/s12906-017-1625-3, 2-s2.0-85012074003. PMC5301383 28183311

[30] Perianayagam J. B. , Narayanan S. , Gnanasekar G. et al., Evaluation of Antidiarrheal Potential of Emblica Officinalis, Pharmaceutical Biology. (2005) 43, no. 4, 373–377, 10.1080/13880200590951856, 2-s2.0-22644446005. 28925835

[31] Tadesse E. , Engidawork E. , Nedi T. , and Mengistu G. , Evaluation of the Anti-diarrheal Activity of the Aqueous Stem Extract of Lantana camara Linn (Verbenaceae) in Mice, BMC Complementary and Alternative Medicine. (2017) 17, no. 1, 10.1186/s12906-017-1696-1, 2-s2.0-85017029187. PMC5379525 28376868

[32] Sini J. M. , Umar I. A. , Anigo K. M. , Stantcheva I. , Bage E. N. , and Mohammed R. , Antidiarrhoeal Activity of Aqueous Extract of Combretum sericeum Roots in Rats, African Journal of Biotechnology. (2008) 7, no. 17.

[33] Rahman M. M. , Islam A. M. , Chowdhury M. A. , Uddin M. E. , and Jamil A. , Antidiarrheal Activity of Leaves Extract of Microcos paniculata Linn in Mice, Int J Pharm. (2012) 2, no. 1, 21–25.

[34] Xu Y. , Zhang H. , and Liu X. W. , Antimicrobial Carbohydrate-based Macromolecules: Their Structures and Activities, Journal of Organic Chemistry. (2020) 85, no. 24, 15827–15836, 10.1021/acs.joc.0c01597. 33231076

[35] Tagousop C. N. , Tamokou J. D. , Ekom S. E. , Ngnokam D. , and Voutquenne-Nazabadioko L. , Antimicrobial Activities of Flavonoid Glycosides from Graptophyllum Grandulosum and Their Mechanism of Antibacterial Action, BMC Complementary and Alternative Medicine. (2018) 18, no. 1, 10.1186/s12906-018-2321-7, 2-s2.0-85053376415. PMC6139119 30219066

[36] Yan Y. , Li X. , Zhang C. , Lv L. , Gao B. , and Li M. , Research Progress on Antibacterial Activities and Mechanisms of Natural Alkaloids: a Review, Antibiotics. (2021) 10, no. 3, 10.3390/antibiotics10030318. PMC8003525 33808601

[37] Thawabteh A. M. , Ghanem A. W. , AbuMadi S. et al., Antibacterial Activity and Antifungal Activity of Monomeric Alkaloids, Toxins. (2024) 16, no. 11, 10.3390/toxins16110489. PMC11598475 39591244

[38] Shamsudin N. F. , Ahmed Q. U. , Mahmood S. et al., Antibacterial Effects of Flavonoids and Their structure-activity Relationship Study: a Comparative Interpretation, Molecules. (2022) 27, no. 4, 10.3390/molecules27041149. PMC8879123 35208939

[39] Farha A. K. , Yang Q. Q. , Kim G. et al., Tannins as an Alternative to Antibiotics, Food Bioscience. (2020) 38, 10.1016/j.fbio.2020.100751.

[40] Huang J. , Zaynab M. , Sharif Y. et al., Tannins as Antimicrobial Agents: Understanding Toxic Effects on Pathogens, Toxicon. (2024) 247, 10.1016/j.toxicon.2024.107812. 38908527

[41] Vollaro A. , Esposito A. , Antonaki E. et al., Steroid Derivatives as Potential Antimicrobial Agents Against Staphylococcus aureus Planktonic Cells, Microorganisms. (2020) 8, no. 4, 10.3390/microorganisms8040468. PMC7232480 32218320

[42] Zou Y. and Fang W. , Naturally Derived Glycosides with Potential Activity Against Staphylococcus aureus, Current Topics in Medicinal Chemistry. (2021) 21, no. 27, 2500–2512, 10.2174/1568026621666211015091539. 34649487

[43] Hu J. S. , Gelman D. B. , Salvucci M. E. , Chen Y. P. , and Blackburn M. B. , Insecticidal Activity of Some Reducing Sugars Against the Sweet Potato Whitefly, Bemisia tabaci, Biotype B, Journal of Insect Science. (2010) 10, no. 1, 203–222, 10.1673/031.010.20301, 2-s2.0-79551615971. 21268696 PMC3029359

[44] Liang P. , Li J. , Chen W. , Li J. , Yang Q. , and Zhang J. , Application of Natural Bioresources to Sustainable Agriculture: Ac-Glycoside Insecticide Based on N-Acetyl-glucosamine for Regulating Insect Molting of Ostrinia furnacalis, Journal of Agricultural and Food Chemistry. (2023) 71, no. 14, 5496–5506, 10.1021/acs.jafc.2c08760. 37013678

[45] Hikal W. M. , Baeshen R. S. , and Said-Al Ahl H. A. , Botanical Insecticide as Simple Extractives for Pest Control, Cogent Biology. (2017) 3, no. 1.

[46] Pereira V. , Figueira O. , and Castilho P. C. , Flavonoids as Insecticides in Crop Protection—a Review of Current Research and Future Prospects, Plants. (2024) 13, no. 6, 10.3390/plants13060776. PMC10975847 38592833

[47] El-Aswad A. F. , Aisu J. , and Khalifa M. H. , Biological Activity of Tannins Extracts from Processed Camellia sinensis (Black and Green Tea), Vicia faba and Urtica dioica and Allium cepa Essential Oil on Three Economic Insects, Journal of Plant Diseases and Protection. (2023) 130, no. 3, 495–508, 10.1007/s41348-022-00680-x.

[48] Ma S. , Jiang W. , Hu Y. , Wang Q. , Wu W. , and Shi B. , Synthesis, Crystal Structure, and Insecticidal Activity of Steroidal N-piperidone, Journal of Agricultural and Food Chemistry. (2022) 70, no. 5, 1467–1476, 10.1021/acs.jafc.1c06075. 35080386

[49] Abbassy M. A. , Abdelgaleil S. A. , Belal A. S. , and Rasoul M. A. , Insecticidal, Antifeedant and Antifungal Activities of Two Glucosides Isolated from the Seeds of Simmondsia chinensis, Industrial Crops and Products. (2007) 26, no. 3, 345–350, 10.1016/j.indcrop.2007.04.005, 2-s2.0-34548204274.

[50] Dubreuil J. D. , Antibacterial and Antidiarrheal Activities of Plant Products Against Enterotoxinogenic Escherichia coli, Toxins. (2013) 5, no. 11, 2009–2041, 10.3390/toxins5112009, 2-s2.0-84887569819. 24212181 PMC3847712

[51] Xie G. , Deng N. , Zheng T. , Peng X. , Zhang S. , and Tan Z. , Total Glycosides Contribute to the Anti-diarrheal Effects of Qiwei Baizhu Powder via Regulating Gut Microbiota and Bile Acids, Frontiers in Cellular and Infection Microbiology. (2022) 12, 10.3389/fcimb.2022.945263. PMC9444044 36072221

[52] Sharma D. K. , Gupta V. K. , Kumar S. et al., Evaluation of Antidiarrheal Activity of Ethanolic Extract of Holarrhena antidysenterica Seeds in Rats, Veterinary World. (2015) 8, no. 12, 1392–1395, 10.14202/vetworld.2015.1392-1395, 2-s2.0-84954228526. 27047049 PMC4774815

[53] Alhumaydhi F. A. , Rauf A. , Rashid U. et al., In Vivo and in Silico Studies of Flavonoids Isolated from Pistacia integerrima as Potential Antidiarrheal Agents, ACS Omega. (2021) 6, no. 24, 15617–15624, 10.1021/acsomega.1c00298. 34179606 PMC8223227

[54] Salama-Müller A. and Roese N. , Antidiarrheal Properties of the Combination of Tannin Albuminate and Ethacridine lactate-a Narrative Review, Natural Product Communications. (2023) 18, no. 5, 10.1177/1934578x231170998.

[55] Rahman M. M. , Ahmad S. H. , Mohamed M. T. , and Ab Rahman M. Z. , Antimicrobial Compounds from Leaf Extracts of Jatropha curcas, Psidium guajava, and Andrographis paniculata, The Scientific World Journal. (2014) 2014, no. 1, 1–8, 10.1155/2014/635240, 2-s2.0-84933057054. PMC4163420 25250382

[56] Linton R. E. , Jerah S. L. , and Bin Ahmad I. , The Effect of Combination of Octadecanoic Acid, Methyl Ester and Ribavirin Against Measles Virus, International Journal Science Technology Research. (2013) 2, no. 10, 181–184.

[57] Dos Reis C. M. , da Rosa B. V. , da Rosa G. P. et al., Antifungal and Antibacterial Activity of Extracts Produced from Diaporthe schini, Journal of Biotechnology. (2019) 294, 30–37, 10.1016/j.jbiotec.2019.01.022, 2-s2.0-85061695414. 30769000

[58] Balachandar R. , Navaneethan R. , Biruntha M. , Kumar K. K. , Govarthanan M. , and Karmegam N. , Antibacterial Activity of Silver Nanoparticles Phytosynthesized from Glochidion candolleanum Leaves, Materials Letters. (2022) 311, 10.1016/j.matlet.2021.131572.

[59] Shah R. and Verma P. K. , Therapeutic Importance of Synthetic Thiophene, Chemistry Central Journal. (2018) 12, no. 1, 10.1186/s13065-018-0511-5, 2-s2.0-85058783112. PMC6768136 30564984

[60] Nagoor Meeran M. F. , Javed H. , Al Taee H. , Azimullah S. , and Ojha S. K. , Pharmacological Properties and Molecular Mechanisms of Thymol: Prospects for Its Therapeutic Potential and Pharmaceutical Development, Frontiers in Pharmacology. (2017) 8, 10.3389/fphar.2017.00380, 2-s2.0-85021671331. PMC5483461 28694777

[61] Dembitsky V. M. , Naturally Occurring Bioactive Cyclobutane-containing (CBC) Alkaloids in Fungi, Fungal Endophytes, and Plants, Phytomedicine. (2014) 21, no. 12, 1559–1581, 10.1016/j.phymed.2014.07.005, 2-s2.0-84907224347. 25442265

[62] Küçük H. B. , Yusufoğlu A. , Mataracı E. , and Döşler S. , Synthesis and Biological Activity of New 1,3-dioxolanes as Potential Antibacterial and Antifungal Compounds, Molecules. (2011) 16, no. 8, 6806–6815, 10.3390/molecules16086806, 2-s2.0-80052220071. 21832971 PMC6264465

[63] Shaaban M. T. , Ghaly M. F. , and Fahmi S. M. , Antibacterial Activities of Hexadecanoic Acid Methyl Ester and Green‐Synthesized Silver Nanoparticles Against Multidrug‐Resistant Bacteria, Journal of Basic Microbiology. (2021) 61, no. 6, 557–568, 10.1002/jobm.202100061. 33871873

[64] Misra D. , Ghosh N. N. , Mandal M. et al., Anti-Enteric Efficacy and Mode of Action of Tridecanoic Acid Methyl Ester Isolated from Monochoria Hastata (L.) Solms Leaf, Brazilian Journal of Microbiology. (2022) 53, no. 2, 715–726, 10.1007/s42770-022-00696-3. 35149984 PMC9151942

[65] Nabi M. , Tabassum N. , and Ganai B. A. , Phytochemical Screening and Antibacterial Activity of Skimmia anquetilia NP Taylor and Airy Shaw: a First Study from Kashmir Himalaya, Frontiers in Plant Science. (2022) 13, 10.3389/fpls.2022.937946. PMC9412939 36035710

[66] Ghareeb M. A. , Hamdi S. A. , Fol M. F. , and Ibrahim A. M. , Chemical Characterization, Antibacterial, Antibiofilm, and Antioxidant Activities of the Methanolic Extract of Paratapes undulatus Clams (Born, 1778), Journal of Applied Pharmaceutical Science. (2022) 12, no. 5, 219–228.

[67] Taher M. A. , Laboni A. A. , Shompa S. A. et al., Bioactive Compounds Extracted from Leaves of G. cyanocarpa Using Various Solvents in Chromatographic Separation Showed Anti-cancer and Anti-microbial Potentiality in in Silico Approach, Chinese Journal of Analytical Chemistry. (2023) 51, no. 12, 10.1016/j.cjac.2023.100336.

[68] Willie P. , Uyoh E. A. , and Aikpokpodion P. O. , Gas chromatography-mass Spectrometry (GC-MS) Assay of bio-active Compounds and Phytochemical Analyses in Three Species of Apocynaceae, Pharmacognosy Journal. (2021) 13, no. 2, 383–392, 10.5530/pj.2021.13.49.

[69] Ghaneian M. T. , Ehrampoush M. H. , Jebali A. , Hekmatimoghaddam S. , and Mahmoudi M. , Antimicrobial Activity, Toxicity and Stability of Phytol as a Novel Surface Disinfectant, Environmental Health Engineering and Management Journal. (2015) 2, no. 1, 13–16.

[70] Lingfa L. and Ankanagari S. , GC-MS Profiling of Reproductive Stage Withania somnifera for Antimicrobial and Anticancer Phytochemicals, Biomedical and Pharmacology Journal. (2023) 16, no. 1, 197–211, 10.13005/bpj/2601.

[71] Zhang K. , Li T. , Shan X. , Lu R. , Zhang S. , and Xu H. , Cholesterol: Bioactivities, Structural Modification, Mechanisms of Action, and structure-activity Relationships, Mini Reviews in Medicinal Chemistry. (2021) 21, no. 14, 1830–1848, 10.2174/1389557521666210105123320. 33402086

[72] Mazumder K. , Nabila A. , Aktar A. , and Farahnaky A. , Bioactive Variability and in Vitro and in Vivo Antioxidant Activity of Unprocessed and Processed Flour of Nine Cultivars of Australian Lupin Species: a Comprehensive Substantiation, Antioxidants. (2020) 9, no. 4, 10.3390/antiox9040282. PMC7222189 32230703

[73] Marini E. , Magi G. , Mingoia M. , Pugnaloni A. , and Facinelli B. , Antimicrobial and Anti-virulence Activity of Capsaicin Against erythromycin-resistant, cell-invasive Group A Streptococci, Frontiers in Microbiology. (2015) 6, 10.3389/fmicb.2015.01281, 2-s2.0-84949803835. PMC4643145 26617603

[74] Kumar A. , Brief Review on Cyclopropane Analogs: Synthesis and Their Pharmacological Applications, International Journal of Pharmacy Science. (2013) 5, no. 1, 467–472.

[75] Ram V. J. , Sethi A. , Nath M. , and Pratap R. , The Chemistry of Heterocycles: Nomenclature and Chemistry of Three to Five Membered Heterocycles, The Chemistry of Heterocycles. (2019) 149–478.

[76] Neoh T. L. , Tanimoto T. , Ikefuji S. , Yoshii H. , and Furuta T. , Improvement of Antifungal Activity of 10-undecyn-1-ol by Inclusion Complexation with Cyclodextrin Derivatives, Journal of Agricultural and Food Chemistry. (2008) 56, no. 10, 3699–3705, 10.1021/jf0731898, 2-s2.0-45549094575. 18454543

[77] Kourmouli A. , Valenti M. , van Rijn E. et al., Can Disc Diffusion Susceptibility Tests Assess the Antimicrobial Activity of Engineered Nanoparticles?, Journal of Nanoparticle Research. (2018) 20, no. 3, 10.1007/s11051-018-4152-3, 2-s2.0-85042879258. PMC5834581 29527123

[78] Barbieri R. , Coppo E. , Marchese A. et al., Phytochemicals for Human Disease: an Update on plant-derived Compounds Antibacterial Activity, Microbiological Research. (2017) 196, 44–68, 10.1016/j.micres.2016.12.003, 2-s2.0-85007482517. 28164790

[79] Khameneh B. , Eskin N. M. , Iranshahy M. , and Fazly Bazzaz B. S. , Phytochemicals: a Promising Weapon in the Arsenal Against antibiotic-resistant Bacteria, Antibiotics. (2021) 10, no. 9, 10.3390/antibiotics10091044. PMC8472480 34572626

[80] Jayaprakash A. , Phytochemicals, Antimicrobial and Antioxidant Properties of Annona reticulata Linn, Journal of Artificial Intelligence Research. (2017) 6, no. 6, 90–95.

[81] Lydia D. E. , John S. , Swetha V. K. , and Sivapriya T. , Investigation on the Antimicrobial and Antioxidant Activity of Custard Apple (Annona reticulata) Peel Extracts, Research Journal of Pharmacognosy and Phytochemistry. (2017) 9, no. 4, 241–247, 10.5958/0975-4385.2017.00045.0.

[82] de Souza Barboza T. J. , Ferreira A. F. , de Paula Rosa Ignacio A. C. , and Albarello N. , Antimicrobial Activity of Anonna Mucosa (Jacq.) Grown in Vivo and Obtained by in Vitro Culture, Brazilian Journal of Microbiology. (2015) 46, no. 3, 785–789, 10.1590/s1517-838246320140468, 2-s2.0-84939477247. 26413061 PMC4568882

[83] Silva R. M. , Silva I. D. , Estevinho M. M. , and Estevinho L. M. , Anti-Bacterial Activity of Annona muricata Linnaeus Extracts: a Systematic Review, Food Science and Technology. (2021) 42, 10.1590/fst.13021.

[84] Egbo C. C. , Igboaka D. C. , and Uzor P. F. , Antimicrobial Assay and GC-MS Profile of the Extract of the Endophytic Fungus from Annona muricata (Annonaceae) Leaf, Tropical Journal of Natural Product Research. (2024) 8, no. 4, 7030–7034, 10.26538/tjnpr/v8i3.37.

[85] Lengai G. M. , Muthomi J. W. , and Mbega E. R. , Phytochemical Activity and Role of Botanical Pesticides in Pest Management for Sustainable Agricultural Crop Production, Scientific African. (2020) 7, 10.1016/j.sciaf.2019.e00239.

[86] Rattan R. S. , Mechanism of Action of Insecticidal Secondary Metabolites of Plant Origin, Crop Protection. (2010) 29, no. 9, 913–920, 10.1016/j.cropro.2010.05.008, 2-s2.0-77955095824.

[87] Parthiban E. , Bhuvaragavan S. , Gonzalez-Ortega O. , Janarthanan S. , and Ramanibai R. , Mosquito Larvicidal Activity of Annona reticulata Extract and Its Lethal Impacts on Allelochemicals Detoxifying Enzymes in Wild Population Dengue vector, Aedes aegypti, International Journal of Pest Management. (2024) 70, no. 3, 478–493, 10.1080/09670874.2021.1998723.

[88] Mallick S. , Banerjee R. , and Chandra G. , Mosquito Larvicidal Potential of Ethanol Leaf Extract of the Plant, Annona reticulata L. Against Aedes aegypti L. and Culex quinquefasciatus Say (Diptera: Culicidae), Journal of Mosquito Research. (2015) 5, no. 19.

[89] Malathi S. , Rameshkumar G. , Rengarajan R. L. , Rajagopal T. , Muniasamy S. , and Ponmanickam P. , Phytofabrication of Silver Nanoparticles Using Annona reticulata and Assessment of Insecticidal and Bactericidal Activities, Journal of Environmental Biology. (2019) 40, no. 4, 626–633, 10.22438/jeb/40/4/mrn-934, 2-s2.0-85071930690.

[90] Njoya E. M. , Fewou P. M. , and Niedermeyer T. H. , (Euphorbiaceae): an Overview of Its Botanical Diversity, Traditional Uses, Phytochemistry, Pharmacological Effects and Perspectives Towards Developing Its Plant-based Products, Journal of Ethnopharmacology. (2021) 277. 10.1016/j.jep.2021.114244 34052354

[91] Imam M. Z. , Sultana S. , and Akter S. , Antinociceptive, Antidiarrheal, and Neuropharmacological Activities of Barringtonia acutangula, Pharmaceutical Biology. (2012) 50, no. 9, 1078–1084, 10.3109/13880209.2012.656850, 2-s2.0-84865248437. 22830487

[92] Megersa A. , Dereje B. , Adugna M. , Ayalew Getahun K. , and Birru E. M. , Evaluation of Anti-diarrheal Activities of the 80% Methanol Extract and Solvent Fractions of Maesa lanceolata Forssk (Myrsinaceae) Leaves in Mice, Journal of Experimental Pharmacology. (2023) 15, 391–405, 10.2147/jep.s429403. 37904837 PMC10613406

[93] Jahan S. , Kar A. , Das A. , Chowdhury M. A. , Islam M. S. , and Hasanuzzaman M. , Antidiarrheal and Antimotility Activities of Stem Bark Extracts of Annona reticulata Linn. in Mice Model, Journal of Applied Life Science International. (2019) 20, no. 3, 1–9, 10.9734/jalsi/2019/v20i330082.

